# NF-YB Regulates Spermatogonial Stem Cell Self-Renewal and Proliferation in the Planarian *Schmidtea mediterranea*

**DOI:** 10.1371/journal.pgen.1006109

**Published:** 2016-06-15

**Authors:** Harini Iyer, James J. Collins, Phillip A. Newmark

**Affiliations:** Howard Hughes Medical Institute and Department of Cell and Developmental Biology, University of Illinois at Urbana-Champaign, Urbana, Illinois, United States of America; University of Oxford, UNITED KINGDOM

## Abstract

Gametes are the source and carrier of genetic information, essential for the propagation of all sexually reproducing organisms. Male gametes are derived from a progenitor stem cell population called spermatogonial stem cells (SSCs). SSCs give rise to male gametes through the coordination of two essential processes: self-renewal to produce more SSCs, and differentiation to produce mature sperm. Disruption of this equilibrium can lead to excessive proliferation of SSCs, causing tumorigenesis, or can result in aberrant differentiation, leading to infertility. Little is known about how SSCs achieve the fine balance between self-renewal and differentiation, which is necessary for their remarkable output and developmental potential. To understand the mechanisms of SSC maintenance, we examine the planarian homolog of Nuclear Factor Y-B (NF-YB), which is required for the maintenance of early planarian male germ cells. Here, we demonstrate that NF-YB plays a role in the self-renewal and proliferation of planarian SSCs, but not in their specification or differentiation. Furthermore, we characterize members of the NF-Y complex in *Schistosoma mansoni*, a parasitic flatworm related to the free-living planarian. We find that the function of NF-YB in regulating male germ cell proliferation is conserved in schistosomes. This finding is especially significant because fecundity is the cause of pathogenesis of *S*. *mansoni*. Our findings can help elucidate the complex relationship between self-renewal and differentiation of SSCs, and may also have implications for understanding and controlling schistosomiasis.

## Author Summary

Sexually reproducing organisms require gametes–sperm and eggs–for the perpetuation of life and transmission of genetic information between generations. Male gametes (sperm) arise from a dedicated population of stem cells known as spermatogonial stem cells (SSCs). Identification of factors involved in SSC maintenance has important biomedical implications, including deciphering the etiology of testicular tumors and optimizing fertility treatments. Here we show that a male germ cell-specific homolog of the ubiquitous Nuclear Factor-Y family of transcription factors, NF-YB, is required for the self-renewal and proliferation of SSCs in the freshwater planarian, *Schmidtea mediterranea*. Additionally, we extend our study to the parasitic flatworm *Schistosoma mansoni*, the causative agent of the major neglected tropical disease schistosomiasis and evolutionary cousin of the free-living planarian. We find that there is a loss of proliferating cells in the testes of the parasite when schistosome NF-Y components are inhibited. This observation is significant since the reproductive output of *S*. *mansoni* is the primary cause of the morbidity associated with schistosomiasis. Together, our results establish NF-YB as an important regulator of SSC maintenance, and may open avenues for combating schistosomiasis.

## Introduction

Spermatogenesis is highly prolific, relying on SSCs for continual production of progeny. This prodigious output must employ multiple mechanisms to maintain the fine balance between SSC self-renewal and differentiation. Understanding the mechanisms of SSC maintenance is crucial for the treatment of several physiological and disease conditions. Self-renewal of SSCs without differentiation can result in tumor formation. For instance, seminoma-like growth of undifferentiated spermatogonia is seen upon expression of activated RAS, or overexpression of GDNF, or Cyclins D2 and E1, or BCL6B [1–3]. In contrast, aberrant development and differentiation of spermatozoa, due to insufficient sperm production, inadequate sperm motility, or abnormal sperm morphology, are the principal causes underlying male infertility [4].

The maintenance of germline stem cells is also a key feature behind the fecundity of trematodes such as *Schistosoma mansoni*, a causative agent of schistosomiasis, a disease affecting over 200 million people worldwide. The pathogenicity of schistosomiasis is due to the body’s immune response to eggs laid by adult worms in their human hosts. The spermatogenic output of these parasites is clear from the observation that individuals with schistosomiasis can pass eggs over 30 years after initial infection [5,6]. Thus, in addition to illuminating the causes behind infertility and tumorigenesis, a better understanding of molecules that play a role in the maintenance of SSCs may provide new approaches for preventing and treating schistosomiasis.

One such molecule is a planarian homolog of Nuclear Factor-Y B (NF-YB), which belongs to the NF-Y family of transcription factors [7–9]. The NF-Y complex has been studied in several developmental contexts in *D*. *melanogaster* [10–12], *C*. *elegans* [13], and *D*. *rerio* [14], and a function in germ cells for this gene family has been described in the freshwater planarian *Schmidtea mediterranea* [15]. More recent work has shown that members of this complex also play roles in somatic stem cell maintenance in the asexual strain of *S*. *mediterranea* [16]. In the sexual strain, upon *NF-YB* knockdown, animals initially lost their SSC pool followed by more differentiated male germ cells. After over a month of *NF-YB(RNAi)*, mature sperm were seen in sperm ducts of sexual planarians, and some animals had small testes filled with mostly spermatids and some sperm. Thus, *NF-YB(RNAi)* animals appeared to complete the initial rounds of spermatogenesis, but failed to maintain sperm production over time, possibly due to the loss of SSCs. This phenotype is strikingly similar to that seen in *Plzf* and *TAF4b* mutant mice [17,18]. How NF-YB coordinates the balance between self-renewal and differentiation decisions of SSCs at both cellular and molecular levels needs further exploration.

In this study, we provide a phenotypic characterization of planarian *NF-YB(RNAi)* using new markers to track individual stages of spermatogenesis [19]. Our experiments indicate that in *S*. *mediterranea*, NF-YB does not control germ cell specification or differentiation, but instead promotes self-renewal and proliferation of early germ cells. Interestingly, the *NF-YB(RNAi)* phenotype in the male germline is strikingly similar in both *S*. *mediterranea* and the trematode *S*. *mansoni*. Our findings provide mechanistic insight into the role of NF-YB, show the conserved function of this molecule in the testes of both free-living and parasitic flatworms, and may have implications for combating schistosomiasis.

## Results

### *NF-YB(RNAi)* results in progressive loss of male germ cells in *Schmidtea mediterranea* starting from the stem cell population

**Fig 1:**
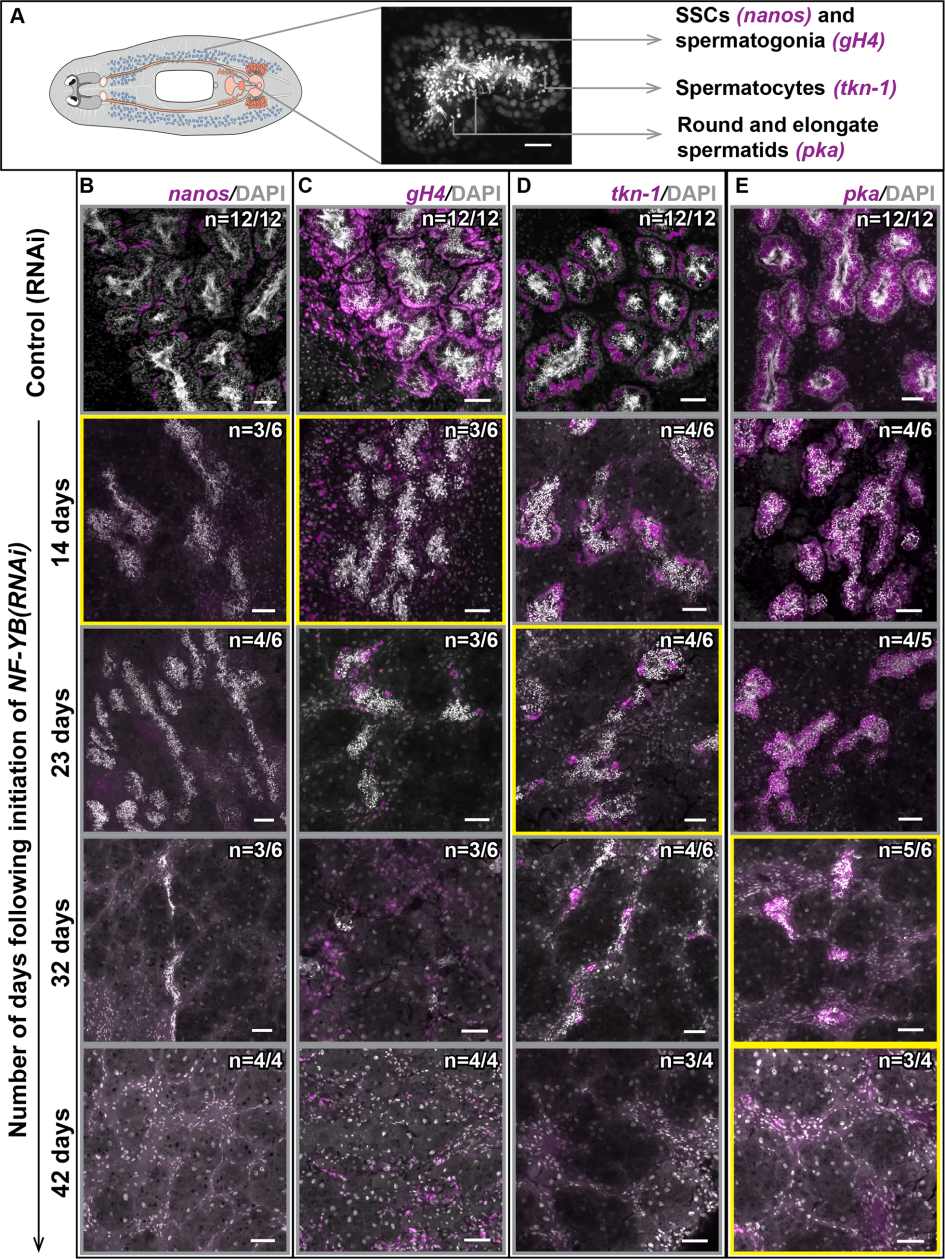
*NF-YB(RNAi)* results in progressive loss of male germ cells in *S*. *mediterranea* starting from the stem cell population. **(A)** Schematic of sexual *S*. *mediterranea* and magnified section of a planarian testis lobe showing the location of individual cell types and their corresponding markers. Scale bar, 20 μm. Control (RNAi) and *NF-YB(RNAi)* animals fixed at 14, 23, 32, and 42 days (4, 6, 8, and 10 feedings of double-stranded RNA (dsRNA), 4–5 days between feedings) following the initiation of RNAi, labeled to detect **(B)** *nanos* (spermatogonial stem cells or SSCs), **(C)** *gH4* (spermatogonia and neoblasts), **(D)** *tkn-1* (spermatocytes), and **(E)** *pka* (spermatids). The increasing severity of *NF-YB(RNAi)* phenotype is evident from the initial loss of the least differentiated male germ cells (SSCs and spermatogonia), followed by the more differentiated male germ cells. The primary cell type being affected at each stage is highlighted with a yellow box. Eventually all male germ cells are lost. The remaining *gH4*^*+*^ cells are neoblasts. The numbers on the figure indicate animals with phenotypes similar to the representative image shown. The remaining animals have either a less severe (similar to the image of the previous RNAi time point) or a more severe (similar to the image of the next RNAi time point) phenotype. Scale bars, 50 μm.

To observe the different stages of *NF-YB(RNAi)* phenotype progression in the male germ cells of *S*. *mediterranea* (schematic Fig 1A), we tracked the following male germ cell populations and their respective signature transcripts: SSCs (= *nanos*), spermatogonia (= *germinal histone H4/gH4*), spermatocytes (= *tektin1/tkn-1*), and spermatids (= *protein kinase A/pka*) [20–22]. Loss of SSCs and spermatogonia was observed at the earliest stages of *NF-YB(RNAi)*, indicated by loss of *nanos* and *gH4* labeling (Figs 1B, 1C and S1). Although the spermatocyte layer was initially unaffected, upon continued knockdown, a reduction in *tkn-1*-labeled spermatocytes was seen (Figs 1D and S1). *NF-YB(RNAi)* animals also show varying degrees of mature spermatozoa loss during the RNAi timecourse. At later time points the testes only contained clusters of spermatids, labeled with *pka* (Figs 1E and S1) and some sperm. Eventually, there was a complete loss of all male germ cells after *NF-YB(RNAi)*. Although all animals showed a progressive loss of male germ cells starting with the least differentiated cells (SSCs and spermatogonia), there was some variability both between samples and within samples in *NF-YB(RNAi)* animals. We hypothesize that this variability could be a reflection of the NF-YB mRNA/protein half-life in the system, or possibly reflect the variability of germ cell turnover among animals and between different testis lobes (S1 Fig, S1 Table).

Since NF-YB is part of a hetero-trimeric complex, requiring its partners NF-YA and NF-YC for transcriptional activation or repression [23–25], we also examined whether other components of the planarian NF-Y complex function in the gonad. We identified and cloned two planarian paralogs of *NF-YA (A1* and *A2)*, two of *NF-YB (B* and *B2)* and one of *NF-YC*. ClustalW analyses showed a high degree of conservation between the histone-fold motifs of these proteins with their human counterparts (S2A Fig). By in situ hybridization, the *NF-YB2* transcript was detected only in somatic cells and excluded from the testes (S2B Fig). *NF-YA1*, *NF-YA2*, and *NF-YC* were detected in the male gonads as well as somatic tissues (S2B Fig). Knockdown of *NF-YB2*, *NF-YA1*, and *NF-YC* resulted in lesions, head regression, and lethality (S2C Fig), suggesting a role for these genes in neoblast (adult somatic stem cells) or somatic maintenance. Our observation is consistent with experiments performed in the asexual strain of *S*. *mediterranea* [16]. Due to the early lethality of these RNAi treatments, we could not ascertain whether these genes also play roles in testes maintenance, and if they phenocopy *NF-YB(RNAi)*. *NF-YA2(RNAi)* had no somatic or germ cell phenotype (S2C and S2D Fig), and its function may be redundant with *NF-YA1*. *NF-YB* appears to be the only subunit of the planarian NF-Y complex with a germline-specific function and this gene belongs to the relatively small group of planarian genes required for early germ cell maintenance. Thus, we directed our focus on its functional characterization.

### NF-YB is not required for the specification of *nanos*-expressing cells in *S*. *mediterranea*

The early germ cell loss seen in *NF-YB(RNAi)* animals is reminiscent of the knockdown of planarian *nanos* [21], a gene with conserved germ cell functions across metazoans (S3 Fig). The similar phenotypes of *NF-YB(RNAi)* and *nanos(RNAi)* led us to speculate that the two genes might act in concert to control germ cell development. The presence of a CCAAT box, the NF-Y DNA binding motif, -118bp upstream of the *nanos* transcription start site made NF-YB an attractive candidate regulator of *nanos* expression.

**Fig 2:**
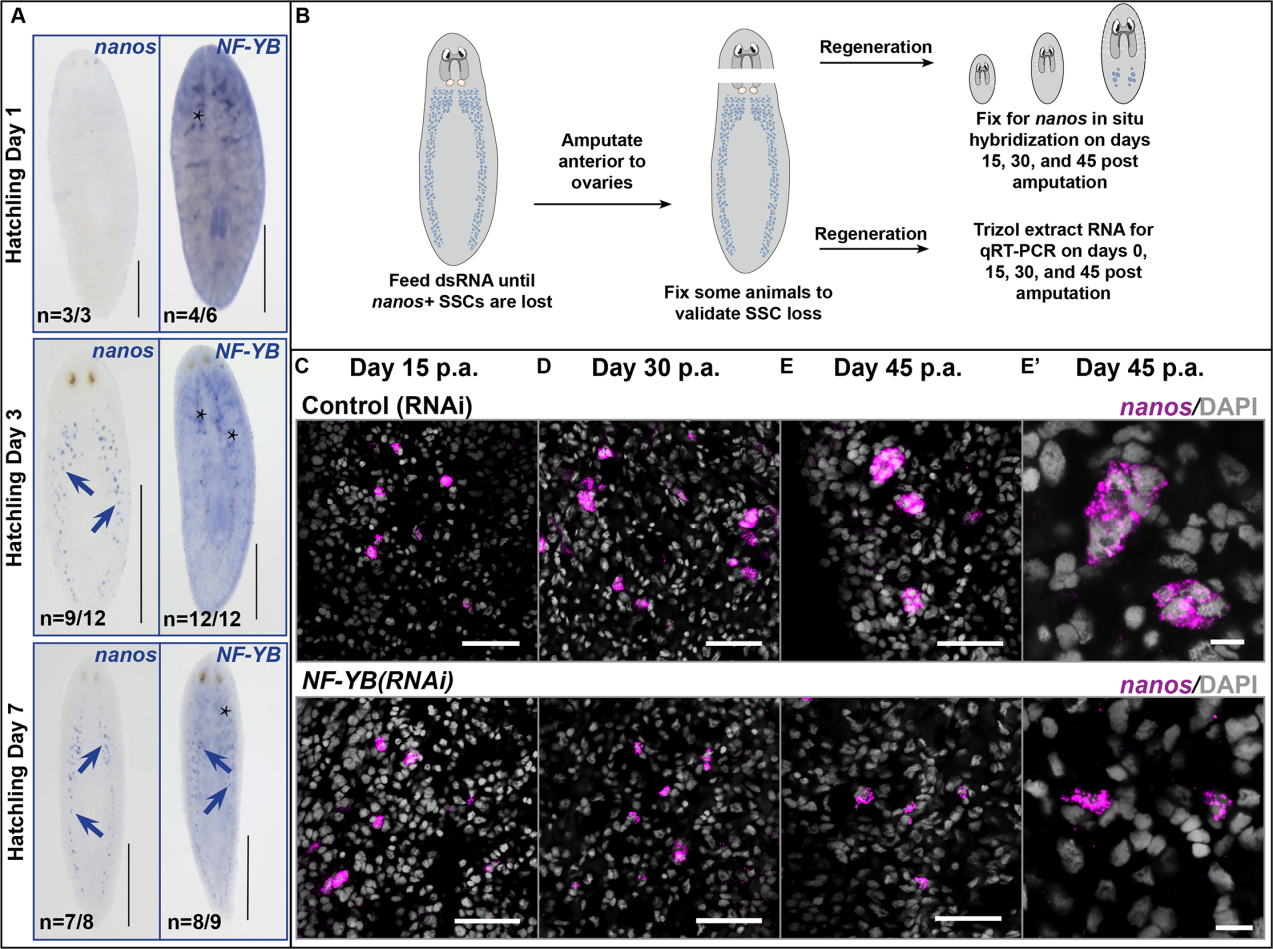
*NF-YB* knockdown does not affect specification of *nanos*-expressing cells in *S*. *mediterranea*. **(A)** *nanos* and *NF-YB* expression at days 1, 3, and 7 following hatching of sexual *S*. *mediterranea*. Blue arrows show presence of SSCs. *NF-YB* expression in germ cells does not precede that of *nanos*. Asterisks show somatic *NF-YB* expression. Scale bars, 0.5 mm. **(B)** Experimental scheme to test if a gene is required for *de novo* specification of *nanos*-expressing cells. **(C)** Fifteen days post amputation, *NF-YB(RNAi)* animals are capable of respecifying *nanos*-expressing cells. On days **(D)** 30 and **(E)** 45 post amputation, *NF-YB(RNAi)* animals do not show clusters of *nanos*^*+*^ SSCs, whereas control (RNAi) animals do. Scale bars, 50 μm. **(E’)** Magnified view of the SSC clusters in 45-day regenerates. Scale bars, 10 μm. p.a.–post amputation.

In *S*. *mediterranea* sexual hatchlings, expression of *nanos* is detected within 3 days post hatching [21]. If NF-YB plays a role in the regulation of *nanos* expression, we reasoned that *NF-YB* expression would precede that of *nanos*. In situ analyses showed that the *NF-YB* transcript was seen in the soma in early hatchlings. However, germline *NF-YB* transcript expression is observed only at later time points relative to *nanos* expression (Fig 2A). This observation suggests that NF-YB does not activate the expression of *nanos*.

Planarians specify germ cells from tissue fragments completely devoid of reproductive structures [21,26,27]. We modified a previously established experimental paradigm [27] to further test whether NF-YB is required for the specification of *nanos*-expressing cells. Briefly, sexually mature planarians were fed *NF-YB* double-stranded (dsRNA) 2–3 times and amputated anterior to the ovaries. The resulting head fragments (lacking reproductive structures at the time of amputation) were monitored for the re-appearance and maintenance of *nanos*-expressing cells at various regeneration time points (Fig 2B).

To ensure that NF-YB protein levels were depleted below the threshold required for the maintenance of *nanos*^*+*^ cells, we performed *NF-YB(RNAi)* in sexually mature planarians (6 feedings over a month) until *nanos*^*+*^ cells were lost (S4A Fig). At fifteen days of regeneration, both control (RNAi) (n = 11/11) and *NF-YB(RNAi)* (n = 11/11) (Fig 2C) head fragments showed *de novo nanos* expression, indicating that *NF-YB* is not required for the respecification of *nanos*^+^ SSCs. There was no significant difference in the number of respecified *nanos*^*+*^ cells between control and *NF-YB(RNAi)* animals at this early time point (n = 11/11 for both, S5A Fig). The respecified *nanos*^*+*^ cells in *NF-YB(RNAi)* animals persisted through regeneration for over a month (Fig 2D–2E’). In later stages of regeneration (45 days post amputation), control animals had numerous *nanos*^*+*^ clusters and many *nanos*^*+*^ cells per cluster, indicating proliferation of SSCs (n = 10/10, Figs 2C–2E’, S5B and S5C). By contrast, *NF-YB(RNAi)* animals had fewer SSC clusters and the *nanos*^*+*^ cells remained mostly as single cells in these clusters (n = 10/10, Figs 2C–2E’, S5B and S5C). *nanos* transcripts in male germ cells were not detected in *dmd1(RNAi)* animals (S4B Fig), consistent with the previously reported role for this gene in SSC specification [24]. We validated the effectiveness and specificity of *NF-YB* knockdown with quantitative real time PCR and in situ hybridization experiments to ensure that *nanos* expression in *NF-YB* knockdown animals was not due to residual *NF-YB*, defective regeneration, or off-target effects (S4C and S6 Figs). Together, these data suggest that *NF-YB* is not required for SSC specification, but may function later in SSC self-renewal or proliferation.

### NF-YB is required for the proliferation of planarian SSCs and spermatogonia in *S*. *mediterranea*

**Fig 3:**
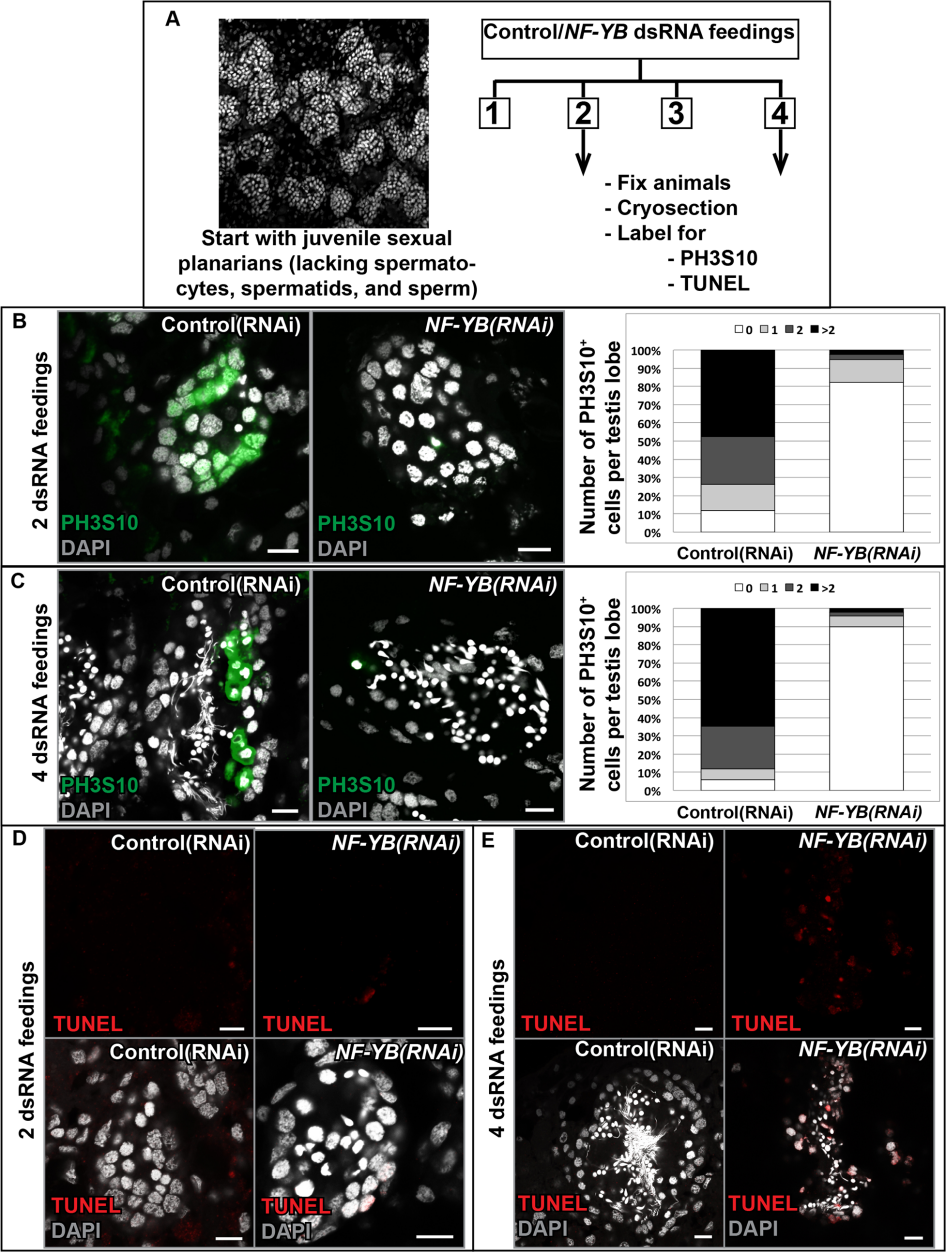
NF-YB is required for proliferation of planarian SSCs and spermatogonia. **(A)** Experimental scheme. **(B)** Cryosections of control and *NF-YB* RNAi animals stained for PH3S10 after 2 feedings and quantification of the results, showing a reduction in mitotic cells. **(C)** This effect is greater following 4 feedings due to the loss of spermatogonial layer. A minimum of 15 testis lobes from 7–8 animals were counted per time point. Raw data are presented in S1 Table. **(D)** There was little TUNEL signal in control and *NF-YB(RNAi)* animals after 2 feedings. **(E)** Following 4 feedings, the differentiated cells in knockdown animals show increased apoptosis compared to controls. See S1 Table for raw data. Scale bars, 10 μm.

The NF-Y complex is associated with cell cycle regulation in other systems [28–32], and is enriched in many stem cell populations [33–37]. After ruling out a role for NF-YB in SSC specification, we tested if NF-YB is required for cell cycle progression of SSCs and spermatogonia and whether early germ cell loss in *NF-YB* knockdown was through differentiation or apoptosis. We knocked down *NF-YB* in juvenile sexual planarians; in these animals, the testes contain clusters of SSCs and spermatogonia, but lack the more differentiated germ cells. Thus, aberrations in spermatogonial differentiation are more easily assayed in these animals compared to mature sexual animals that already possess differentiated male germ cells. The dsRNA-fed animals were processed at early and late knockdown timepoints, cryosectioned, and sections of the same animal were used for phospho-histone H3 (PH3S10) and TUNEL labeling (Fig 3A).

In planarian testes, the SSCs give rise to spermatogonia, undergoing three rounds of mitosis with incomplete cytokinesis, which can be easily visualized by PH3S10 staining [38,39]. *NF-YB(RNAi)* animals showed a dramatic reduction in mitotic cell number following two feedings of dsRNA (Fig 3B and S1 Table). We confirmed that *NF-YB(RNAi)* animals do not show a reduction in *nanos*^*+*^ SSCs or the *nanos* transcript at this RNAi timepoint (S7 Fig). Not surprisingly, this difference was more pronounced after four feedings, following loss of the mitotic spermatogonial layer (Fig 3C and S1 Table). Our analysis clearly indicates a reduction in proliferation of SSCs and spermatogonia upon *NF-YB* knockdown.

We next tested whether germ cells were undergoing apoptosis in *NF-YB(RNAi)* animals. We found low levels of TUNEL labeling in *NF-YB(RNAi)* animals after two feedings of dsRNA (Fig 3D, S1 Table). However, following four feedings of *NF-YB* dsRNA there was an increase in apoptosis of the differentiated male germ cells (Fig 3E, S1 Table). *NF-YB(RNAi)* animals in later stages of RNAi (after four feedings) still have differentiated male germ cells (spermatocytes and spermatids), indicating that the early germ cells are unlikely to be undergoing apoptosis themselves and are capable of differentiation. Conditional deletion of both *NF-YB* alleles in primary mouse embryonic fibroblasts causes a block in progression of the cell cycle and induction of apoptosis [40]. We speculate that a similar mechanism of NF-YB-mediated testis-maintenance could be acting here.

### *Sm-NF-YB(RNAi)* results in fewer proliferating male germ cells in the parasite *Schistosoma mansoni*

**Fig 4:**
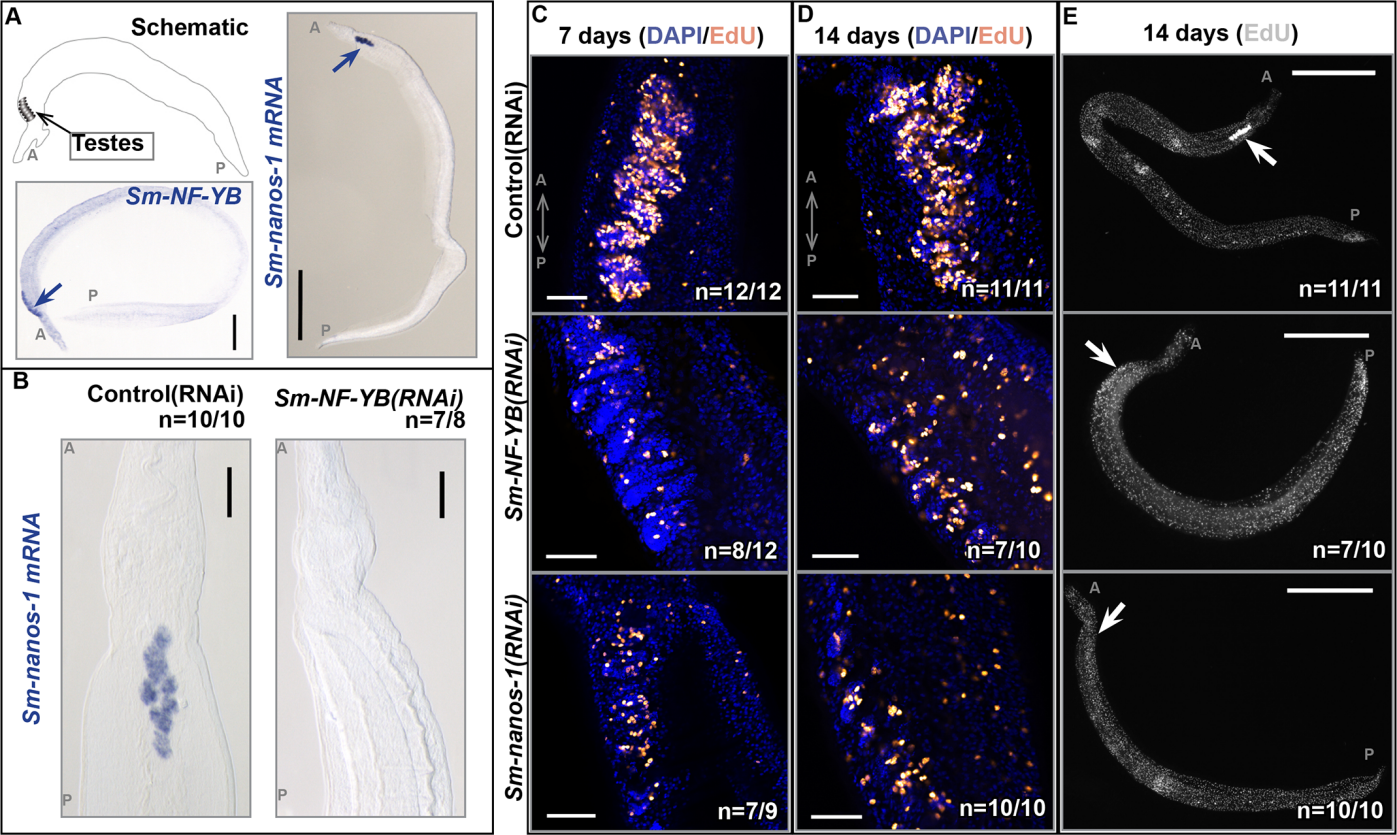
*Sm-NF-YB(RNAi)* results in fewer proliferating male germ cells in the parasite *S*. *mansoni*. **(A)** Illustration of male *S*. *mansoni* depicting the location of the testes and whole-mount in situ hybridization (WISH) in male schistosomes showing *Sm-NF-YB* and *Sm-nanos-1* expression in testes. **(B)** Magnified view of the *Sm-nanos-1* expression in control (RNAi) and *Sm-NF-YB(RNAi)* animals. *Sm-nanos-1* expression is not detected in *Sm-NF-YB(RNAi)* animals. Scale bars, 1 mm. **(C-E)** EdU labeling of control (RNAi), *Sm-NF-YB(RNAi)*, and *Sm-nanos-1(RNAi)* in male schistosomes. **(C)** There are fewer proliferating cells in the testes at early knockdown time points in *Sm-NF-YB(RNAi)* and *Sm-nanos-1(RNAi)* animals. Scale bars, 50 μm. **(D)** At later stages of *Sm-NF-YB(RNAi)*, the testis structure disintegrates, most likely due to the loss of cycling male germ cells. Scale bars, 50 μm. **(E)** Whole-mount animals at later stages of RNAi to show distribution of proliferating cells. There are few or no proliferating cells in the testes of *Sm-NF-YB(RNAi)* and *Sm-nanos-1(RNAi)* animals. Scale bars, 1 mm.

We have previously shown that molecular similarities exist between planarian and schistosome somatic stem cells [41–43]; however, similarities between the germ cells of these two flatworms remain unexplored. To test if the NF-Y complex plays a similar role in the gonads of free-living and parasitic flatworms, we examined the role of NF-Y components in the parasite *S*. *mansoni*. We were especially interested in this comparison because the morbidity associated with schistosomiasis is a result of the tremendous reproductive output of the parasite. Inhibiting fertilization or propagation by blocking germ cell production may open novel avenues for treating this disease. Although schistosomes are dioecious, we restricted our analysis to male schistosomes (Schematic in Fig 4A) due to the testis-specific function of NF-YB in planarians [15].

In situ hybridization revealed that *Sm-NF-YB*, *Sm-NF-YA*, and *Sm-NF-YC* (Figs 4A and S8A) were enriched in the parasite testes, with possible low levels of somatic expression. Further, we found that *Sm-nanos-1* is expressed in the testes of *S*. *mansoni* (Fig 4A). Next, we asked if NF-YB plays a similar role in schistosome testis maintenance. We knocked down the NF-Y complex components in schistosomes by culturing the worms in vitro in the presence of dsRNA [41]. Similar to the planarian *NF-YB* knockdown phenotype, we found that following seven days of dsRNA treatment, *Sm-NF-YB(RNAi)* animals, and surprisingly *Sm-NF-YA(RNAi)* and *Sm-NF-YC(RNAi)* animals, showed a loss of *Sm-nanos-1* labeling (Figs 4B and S8B).

To determine whether loss of *nanos* expression is due to reduced proliferation of the male germ cells, we performed a 24-hour EdU pulse in vitro at early (7 days) and late (14 days) RNAi time points. After 7 days of knockdown, control (RNAi) animals showed a large number of EdU^+^ cells in the testes (Fig 4C). In contrast, *Sm-NF-YB(RNAi)* animals had fewer EdU^+^ male germ cells (Fig 4C), while maintaining an intact testis structure (assessed using DAPI labeling). Similarly, *Sm-nanos-1(RNAi)* animals had fewer proliferating male germ cells but greater male germ cell loss compared to *Sm-NF-YB(RNAi)* animals (Fig 4C). Following 14 days of RNAi, most male germ cells were lost in both *Sm-NF-YB(RNAi)* and *Sm-nanos-1(RNAi)* animals (Fig 4D and 4E). *Sm-NF-YA* and *Sm-NF-YC* knockdown animals showed a similar RNAi phenotype (S8C Fig). Together, we conclude that the role of NF-YB in male germ cell proliferation is conserved in both free-living and parasitic flatworms.

## Discussion

Significant progress has been made in understanding post-transcriptional regulation and the role of RNA-binding proteins in the germline [44–46]. However, mechanisms of transcriptional regulation in germ cells have received relatively less attention. A variant of TFIIA, ALF or TFIIAτ, is expressed in male and female gonads in mice and *Xenopus*, and can substitute for TFIIA in core promoters [47,48]. A TATA-Binding Protein (TBP) variant, TLF/TRF2 is not required for the female reproductive system, but loss of TLF/TRF2 results in an inability to complete spermatogenesis in males [49–51]. Several TBP-associated factors (TAFs) have germ cell-specific roles. Deletion of TAF4b in both female [52] and male [19] gonads results in sterility. A TAF-II-80 homolog, *cannonball*, is expressed in *Drosophila* spermatocytes, and mutations in this gene block spermatid differentiation [53]. Here, we studied a planarian male germ cell-specific transcription factor, NF-YB, exploring the cellular mechanisms of NF-YB-mediated maintenance of early planarian male germ cells. We also found gonadal enrichment of NF-Y components in male schistosomes, and a requirement for *NF-Y* genes in the proliferation and maintenance of male germ cells in these parasites.

Members of the NF-Y complex are enriched in stem cell populations in many other systems. In proliferating skeletal muscle cells the NF-Y complex, and its target cyclin B1, are expressed at high levels; however, in terminally differentiated cells there is a loss or reduction in NF-Y components [33]. In hematopoietic stem cells the NF-Y complex activates HOXB4, a homeobox gene that is expressed abundantly in primitive HSCs. During HSC differentiation there is a decline in NF-Y binding to the HOXB4 promoter and a concomitant reduction in *HOXB4* transcript levels [54]. Subsequent work showed that NF-Ya overexpression in HSCs shifts the balance towards HSC-self-renewal rather than differentiation [34]. By contrast, deletion of *NF-Ya* caused an accumulation of HSCs in G2/M phase of the cell cycle, followed by apoptosis, possibly as a result of dysregulation of key genes involved in cell cycle control, apoptosis, and self-renewal [36]. In human embryonic stem cells (ESCs), the NF-Y complex is required for proliferation and isoforms of NF-Ya are differentially expressed during differentiation [35]. A recent study showed that, in addition to its housekeeping functions, the NF-Y complex regulates ESC identity by coordinating the binding of ESC master transcription factors to core self-renewal and pluripotency genes [37]. Our finding that planarian NF-YB is necessary for self-renewal and proliferation of SSCs and spermatogonia is consistent with the known functions of the NF-Y complex in these other stem cell systems, and provides insight into the role of this transcription factor family in germ cells. We also performed functional characterization of the NF-Y complex in the parasite *Schistosoma mansoni*. Previous work reported that the *S*. *mansoni* NF-YA protein is expressed in both male and female gonads and levels of Sm-NF-YA decreased as maturation of the male germ cells progressed [55]. We found that NF-Y components are necessary for the proliferation of male germ cells in *S*. *mansoni*.

The *NF-YB(RNAi)* phenotype in planarians is strikingly similar to those observed in *Plzf* [17,18] and *TAF4b* [19] mutant mice, both of which undergo progressive loss of spermatogonia with age. This progressive loss, from the least differentiated to the most differentiated germ cells, is not expected in the case of meiotic or maturation defects, strongly indicating that all three genes function in early male germ cell maintenance. Both *Plzf* and *TAF4b* mutant mice are born with normal number of gonocytes/primordial germ cells, indicating proper specification of the germ cells; planarian tissue fragments lacking germ cells and *NF-YB* activity regenerate normal number of SSCs, consistent with proper specification of these cells. *Plzf* and *TAF4b* mutant mice complete the initial rounds of spermatogenesis but show decreasing fertility with age; in planarians, *NF-YB(RNAi)* SSCs and spermatogonia are able to differentiate initially but fail to do so over time. *Plzf* and *TAF4b* mutant mice show a decrease in proliferative spermatogonia over time; *NF-YB(RNAi)* animals also show reduced proliferation of male germ cells in both *S*. *mediterranea* and *S*. *mansoni*. Given the striking similarities between Plzf, TAF4b, and NF-YB functions in the male gonad, it is not unreasonable to speculate that these three genes might be controlling similar targets, or genes that perform similar functions.

We also observed an increase in apoptotic germ cells in later stages of *NF-YB(RNAi)*. *NF-YB(RNAi)* results in loss of *nanos*^*+*^ SSCs, but these cells do not show obvious TUNEL labeling. Several possibilities exist to explain this observation. SSCs may be undergoing apoptosis but the signal may be too weak or transient to be detected, or they may use a non-apoptotic mechanism of cell death. It is also possible that the early germ cells could be entering the differentiation pathway aberrantly, resulting in apoptosis of the differentiating cells. *NF-YB(RNAi)* results in loss of elongated spermatids and sperm in addition to early germ cells (Figs 1 and S1). Although the primary phenotype of *NF-YB(RNAi)* is the loss of early germ cells, the expression of *NF-YB* transcript in the more differentiated male germ cells leaves open the possibility that NF-YB regulates additional target(s) vital for the survival of these differentiated cells.

The identification of a testis-specific component of the planarian NF-Y complex, and our finding that *NF-YB* is required for the maintenance of planarian SSCs, provides a valuable tool for understanding the dynamics of early, undifferentiated germ cells. Putative SSC-specific targets of NF-YB will help reveal the function of the NF-Y complex in the planarian, in which tissue-specific knockdown is not possible. Several genes required for the maintenance of various stages of planarian germ cell development have homologs in other species [15]. Many of these genes, such as *rap55*, *ELAV*, and *MSY4* [56–60], are known to play roles in germ cell development in vertebrates. Given the observation that the *NF-YB* transcript is upregulated in mouse spermatogonia relative to other male germ cells [59], we predict that the role of NF-YB in SSC self-renewal and proliferation might be conserved in vertebrates. Thus, a better understanding of SSC-specific NF-YB targets in planarians is expected to yield insight into the workings of early germ cells across different systems.

## Materials and Methods

### Ethics statement

In adherence to the Animal Welfare Act and the Public Health Service Policy on Humane Care and Use of Laboratory Animals, all experiments with and care of vertebrate animals were performed in accordance with protocols approved by the Institutional Animal Care and Use Committee (IACUC) of the University of Illinois at Urbana-Champaign (protocol approval number 13017).

### Planarian culture

Clonal lines of hermaphroditic *S*. *mediterranea* [20] were maintained in 0.75X Montjuïc salts at 18°C [61]. Clonal asexual lines [62] were maintained in 0.5 g/L Instant Ocean Sea Salts at 20°C.

### Cloning of NF-Y complex

Coding DNA sequences of the NF-Y complex were obtained from SmedGD [63]. Mixtures of sexual and asexual planarian cDNA were used as templates to clone NF-Y components, using primers in S2 Table.

### qRT-PCR

Total RNA was extracted using TRIzol (Invitrogen) according to manufacturer’s instructions, DNase treated (Fisher Scientific) and purified using an RNA clean-up kit (Zymo) before reverse transcription (iScript, Bio-Rad). Prior to RNA extraction, animals were starved for 7 days after the last RNAi feeding to ensure that any remnant dsRNA was cleared from the system. qPCR was performed using GoTaq qPCR master mix (Promega) using Applied Biosystems StepOne Plus RT-PCR system. All experiments were done in biological and technical triplicates. Transcript levels were normalized to *β-tubulin* (primers in S2 Table). Relative mRNA levels were calculated using ΔΔCT [64].

### In vitro transcription and RNA interference (RNAi)

Planarian double-stranded RNA (dsRNA) synthesis and feeding were performed as previously described [65]. Briefly, dsRNA diluted to 15 μg/ml in 2:1 minced liver:planarian salts was fed to planarians once every 4–5 days. The *ccdb* bacterial gene encoded in pJC53.2 [65] served as the negative control.

### In situ hybridization

Whole-mount in situ hybridization of planarians was performed as previously described [66] with modifications for the sexual strain [21,22,67].

### Imaging

Samples developed through the NBT/BCIP colorimetric method were mounted in 80% glycerol and imaged using a Leica M205A stereomicroscope (Leica, Wetzlar, Germany), equipped with Leica DFC420 camera. Whole-mount FISH animals were mounted in Vectashield (Vector Laboratories, Burlingame, CA) and imaged on a Zeiss Stereo Lumar V12 (Carl Zeiss, Germany). For confocal FISH images, samples were mounted in Vectashield and imaged using a Zeiss LSM710 confocal microscope running ZEN 2011. Images were processed using Adobe Photoshop CS5.

### Immunofluorescence on sections

Planarians were cut longitudinally and one half was killed with 2% HCl for 3 minutes on ice and fixed in Methacarn. Cryosectioning was performed as previously described [68]. Anti-Phospho-histone H3 (Cell Signaling, number: 3377S) was used at 1:500 dilution overnight at 4°C. Secondary antibody (anti-rabbit HRP-Jackson labs 111-035-003) was used at 1:500. DAPI (1 μg/ml) was added to the secondary antibody solution. Tyramide signal amplification was performed using FITC-tyramide (Perkin Elmer). Slides were rinsed in PBSTx and mounted in Vectashield.

### TUNEL on sections

A whole-mount TUNEL protocol [69] was modified for cryosections. Planarians were cut longitudinally and one half was treated with 10% N-acetyl-L-cysteine for 7.5 minutes and fixed in 4% formaldehyde in PBSTx (0.3% Triton X-100) for 20–30 minutes at room temperature. Cryosectioning was done as described previously [68]. Rehydration included treatment with pre-chilled ethanol:acetic acid (2:1) at –20°C for 5 minutes. After equilibration, slides were rinsed twice in DI water, and equilibrated in equilibration buffer (100 mM Tris-HCl pH 7.5 + 1 mg/ml IgG-free BSA). Slides were covered with TdT solution (0.5 μl NEB TdT (Cat. No. M0252L), 2 μl NEB buffer 4, 2 μl 2.5 mM CoCl_2_, 0.8 μl 1:50 DIG-dUTP in dATP, 14.7 μl water). After rinsing 3X with PBSTx, the sections were blocked with 5% Horse Serum (Sigma H1138) in PBSTx for 30 minutes. Block was replaced with 1:1000 anti-DIG-POD (Roche 11207733910) diluted in block solution. DAPI (1 μg/ml) was added at this step. Sections were covered with coverslips and incubated for 1 hour at RT. Signal was revealed using Cy3-tyramide (Perkin-Elmer). Slides were rinsed in PBSTx and mounted in Vectashield.

### *Schistosoma* culture and RNAi

*Schistosoma mansoni*, Strain NMRI—exposed Swiss Webster mice (NR-21963) were provided by the NIAID Schistosomiasis Resource Center at the Biomedical Research Institute (Rockville, MD) through NIH-NIAID Contract HHSN272201000005I for distribution through BEI Resources. Mice were perfused with DMEM containing 10% heat-inactivated serum and schistosomes were cultured in vitro [41]. In situ hybridization was performed as previously described [41]. For RNAi, animals (in quadriplicates) were soaked in dsRNA generated by in vitro transcription (30 μg RNA per 10–12 pairs in 3 ml of Basch 169 medium [70,71]). Animals were incubated for 7 and 14 days at 37°C. EdU pulse chase and detection were performed as described previously [41].

### Accession numbers

Nucleotide sequences have been deposited in GenBank with the following accession numbers: NF-YB—KU366699; NF-YB2—KU366700; NF-YA1—KU366701; NF-YA2—KU366702; NF-YC—KU366703.

## Supporting Information

S1 Fig Quantification of the *NF-YB(RNAi)* phenotype in adult sexual animals. Percentage of testis lobes showing normal expression of each male germ cell marker during different *NF-YB(RNAi)* time points. *nanos*^*+*^ SSCs were the first cells to be lost in *NF-YB(RNAi)* animals, after 14 days of RNAi (4 feedings), closely followed by *gH4*^*+*^ SSCs and spermatogonia. At these early stages, very few testis lobes showed reduced *tkn-1*^*+*^ spermatocytes and *pka*^*+*^ spermatids. By 23 days of RNAi (6 feedings), more testis lobes showed reduced *nanos* and *gH4* expression, and the number of testis lobes with reduced *tkn-1* labeling increased slightly. 32 days after starting RNAi (8 feedings), all testis lobes examined lacked *nanos* and *gH4* labeling, many testis lobes showed reduced *tkn-1* expression and about half the lobes showed reduced *pka* expression. By 42 days (10 feedings) almost all germ cells were lost. Elongated spermatids and sperm were lost early (between 14–23 days, 4–6 feedings) and this loss was visualized using DAPI. Ten testis lobes per animal (n = 4–6) were counted for each testis marker per RNAi time point.
(TIF)

S2 Fig NF-YB belongs to the Nuclear Factor-Y family of transcription factors. **(A)** ClustalW analysis of the human and planarian NF-Y complex members showing the highly conserved domains. **(B)** *NF-YB2* transcript is expressed in somatic tissues. *NF-YA1*, *NF-YA2*, and *NF-YC* are expressed in both the testes and the soma. Scale bars, 1 mm. **(C)** RNAi of *NF-YB2*, *NF-YA1*, or *NF-YC* results in lesions, head regression (shown with arrows), and lethality after 5 feedings of dsRNA spaced 5 days apart. *NF-YA2(RNAi)* animals show no somatic phenotype. **(D)** *NF-YA2(RNAi)* animals show no loss of germ cells following 6 feedings of dsRNA. Scale bars, 50 μm.
(TIF)

S3 Fig *nanos(RNAi)* phenotype. Animals show an initial loss of SSCs and spermatogonia followed by the more differentiated cells of the testes. Animals were fixed following 2, 4, 6, and 8 feedings, with 4–5 day intervals between feedings. There are subtle differences between *NF-YB* and *nanos* knockdown animals. In addition to the loss of early germ cells, *NF-YB(RNAi)* animals also show the loss of mature sperm to varying degrees. After 4 feedings of dsRNA, the most differentiated stage present in *NF-YB(RNAi)* animals is round spermatids. *nanos(RNAi)* animals do not show loss of spermatozoa during the initial stages of RNAi. The *nanos(RNAi)* phenotype also manifests faster. Scale bars, 50 μm.
(TIF)

S4 Fig Validation of *NF-YB(RNAi)* efficacy and specificity. **(A)** Following 6 feedings of dsRNA, *nanos* was not detected in the testes of *NF-YB(RNAi)* animals. **(B)** *dmd1(RNAi)* animals do not respecify their male germ cells. Scale bars, 50 μm. **(C)** qRT-PCR to measure the levels of the *NF-YB* transcript (to determine the efficiency of knockdown), *NF-YB2* transcript (to ensure specificity of *NF-YB* knockdown), and *smedwi1* transcript (to determine if the somatic stem cells/neoblasts are perturbed following *NF-YB* knockdown). RNA extraction was done immediately following amputation (Day 0), and at timepoints when head regenerates were fixed for *nanos* in situ hybridization (Days 15, 30, or 45). Unpaired, parametric two-tailed T-test with Welch’s correction was performed on all samples. *NF-YB(RNAi)* animals showed significant reduction in *NF-YB* mRNA levels (*** = P value 0.0001–0.001; ** = P value 0.001–0.01; * = P value 0.01–0.1; n.s. = not significant).
(TIF)

S5 Fig Quantification of de novo specified SSCs. **(A)** 15 days post amputation (p.a.) control and *NF-YB(RNAi)* animals showed 10.1 ± 1.6 (n = 11/11) and 13.7 ± 2.2 (n = 11/11) SSCs respectively. The difference was not significant. **(B)** 45 days p.a. control animals (56.4 ± 6.2, n = 10/10) showed significantly (P<0.05) higher number of SSC clusters than *NF-YB(RNAi)* animals (26.1 ± 2.7, n = 10/10). **(C)** 45 days p.a., the number of *nanos*^*+*^ cells per SSC cluster was significantly (P<0.05) higher in control animals (3.2 ± 0.2, n = 66 from 10 animals) compared to *NF-YB(RNAi)* animals (1.3 ± 0.1, n = 74 from 10 animals). Scatter plots show mean with SD. Unpaired parametric two-tailed T-test with Welch’s correction was performed on all samples to determine significance (**** = P value <0.0001; *** = P value 0.0001–0.001; n.s. = not significant).
(TIF)

S6 Fig Additional validation *NF-YB(RNAi)* specificity. This experiment was performed to demonstrate that two halves of the *NF-YB* transcript can each knock down *NF-YB* mRNA and *nanos*^*+*^ SSCs are respecified in either knockdown experiment. **(A)** Experimental schematic. The experiment for *de novo* respecification of germ cells was repeated using dsRNA corresponding to the 5’ end of the *NF-YB* coding sequence as template. In situ hybridization was used to detect *NF-YB* and *nanos* mRNAs. A riboprobe corresponding to the 3’ end of *NF-YB* coding sequence was generated and used for FISH. **(B)** Control (RNAi) and *NF-YB-5’(RNAi)* animals show *nanos* expression following regeneration. **(C)** Control (RNAi) animals show expression of *NF-YB*, *NF-YB-5’(RNAi)* animals do not. Bottom panel–low magnification view of the hatchling with additional exposure showing the inability to detect *NF-YB* transcript throughout the animal. **(D-F)** The above experiment was also performed using the 3’ end of the *NF-YB* transcript. Scale bars, 50 μm.
(TIF)

S7 Fig *nanos* expression is unaffected at early *NF-YB(RNAi)* time point. **(A)** Following 2 feedings of dsRNA (n = 6/6), *NF-YB(RNAi)* animals exhibit robust *nanos* labeling. From multiple prior RNAi experiments, we know that loss of *nanos*^*+*^ cells in *NF-YB(RNAi)* animals occurs only following 4–6 feedings of dsRNA. Scale bars, 50 μm. **(B)** We quantified SSCs in control and *NF-YB(RNAi)* animals to ensure that the reduced PH3S10 labeling was not due to fewer *nanos*^*+*^ cells in *NF-YB(RNAi)* animals. Following 2 feedings of dsRNA (6 animals each, 4–8 testis lobes per animal), percentage of *nanos*^*+*^ cells per testis lobe in *NF-YB(RNAi)* animals (28.2 ± 1.4, n = 44) was not significantly different (P<0.05) from control (RNAi) animals (24.8 ± 1.2, n = 42). Unpaired parametric T-test with Welch’s correction was performed. Scatter plot shows mean with SD. **(C)** qRT-PCR assay showing that *nanos* mRNA levels were unaffected following 2 feedings of *NF-YB* dsRNA. Unpaired parametric two-tailed T-test with Welch’s correction was performed to determine significance (* = P value 0.01–0.1; n.s. = not significant).
(TIF)

S8 Fig *Sm-NF-YA(RNAi)* and *Sm-NF-YC(RNAi)* animals have fewer proliferating cells in the testes. **(A)** Illustration of male *S*. *mansoni* depicting the location of the testes and whole-mount in situ hybridization (WISH) in male schistosomes showing *Sm-NF-YA* and *Sm-NF-YC* expression in testes. Scale bars, 1 mm. **(B)** Magnified view of *Sm-nanos-1* expression in control (RNAi), *Sm-NF-YA(RNAi)*, and *Sm-NF-YC(RNAi)* animals. *Sm-nanos-1* expression is not detected in *Sm-NF-YA(RNAi)*, and *Sm-NF-YC(RNAi)* animals. Scale bars, 1 mm. **(C)** Left and middle panels show high magnification view of the testes in control (RNAi), *Sm-NF-YA(RNAi)*, and *Sm-NF-YC(RNAi)* animal at early and late KD time points. Scale bars, 50 μm. Right panel shows whole-mount images showing reduction or loss of EdU labeling in the testes in *Sm-NF-YA(RNAi)* and *Sm-NF-YC(RNAi)* animals. Scale bars, 1 mm.
(TIF)

S1 Table Raw data for quantification performed in the manuscript. (DOCX)

S2 Table Primer sequences. (DOCX)
