# Supplementary figures and images for: NF-YB Regulates Spermatogonial Stem Cell Self-Renewal and Proliferation in the Planarian Schmidtea mediterranea
Source: PLoS Genet. 2016 Jun 15;12(6):e1006109. doi: 10.1371/journal.pgen.1006109 (PMC4909293; doi:10.1371/journal.pgen.1006109)

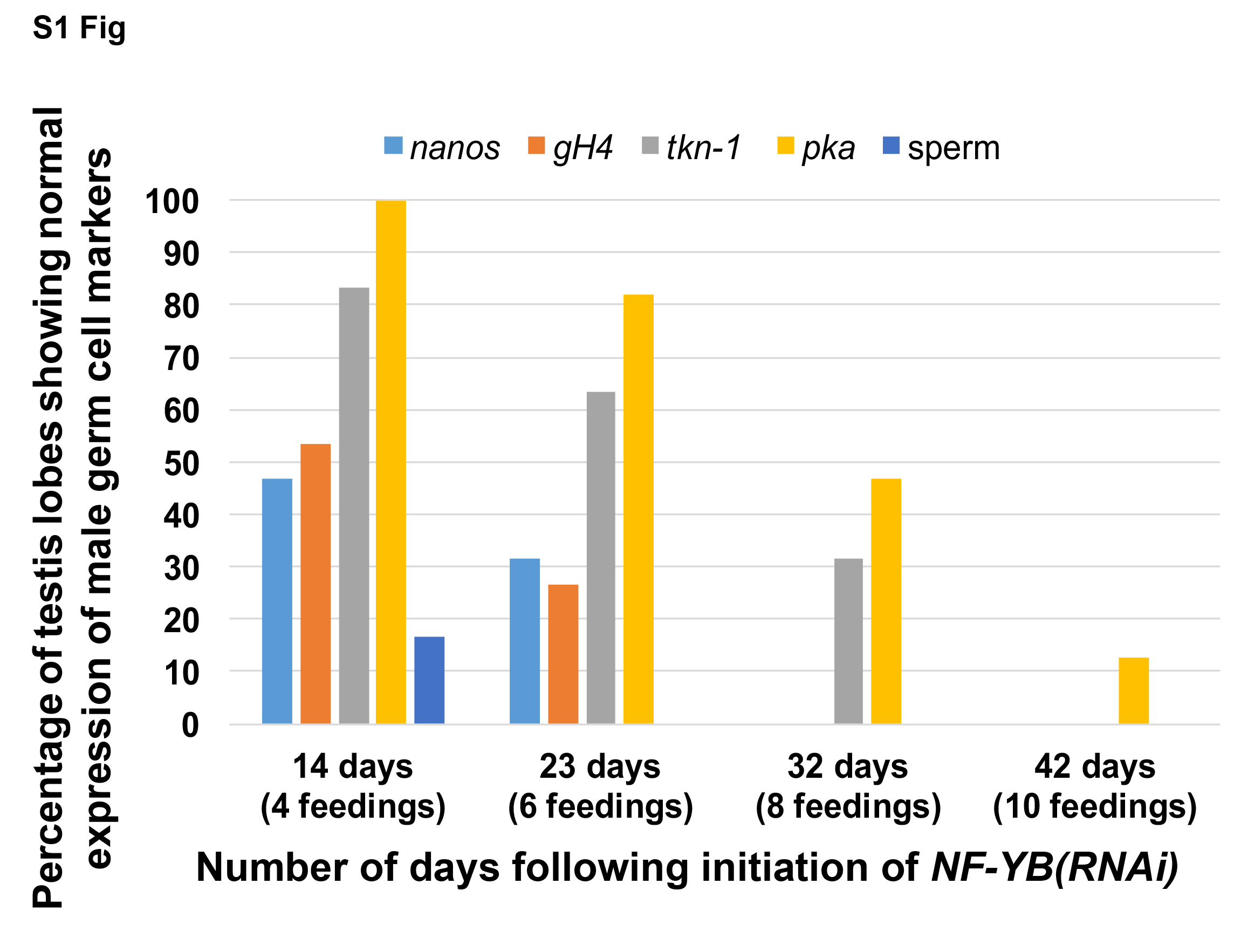

Supplement: S1 Fig — Percentage of testis lobes showing normal expression of each male germ cell marker during different NF-YB(RNAi) time points. nanos+ SSCs were the first cells to be lost in NF-YB(RNAi) animals, after 14 days of RNAi (4 feedings), closely followed by gH4+ SSCs and spermatogonia. At these early stages, very few testis lobes showed reduced tkn-1+ spermatocytes and pka+ spermatids. By 23 days of RNAi (6 feedings), more testis lobes showed reduced nanos and gH4 expression, and the number of testis lobes with reduced tkn-1 labeling increased slightly. 32 days after starting RNAi (8 feedings), all testis lobes examined lacked nanos and gH4 labeling, many testis lobes showed reduced tkn-1 expression and about half the lobes showed reduced pka expression. By 42 days (10 feedings) almost all germ cells were lost. Elongated spermatids and sperm were lost early (between 14–23 days, 4–6 feedings) and this loss was visualized using DAPI. Ten testis lobes per animal (n = 4–6) were counted for each testis marker per RNAi time point. (TIF) [file pgen.1006109.s001.tif]

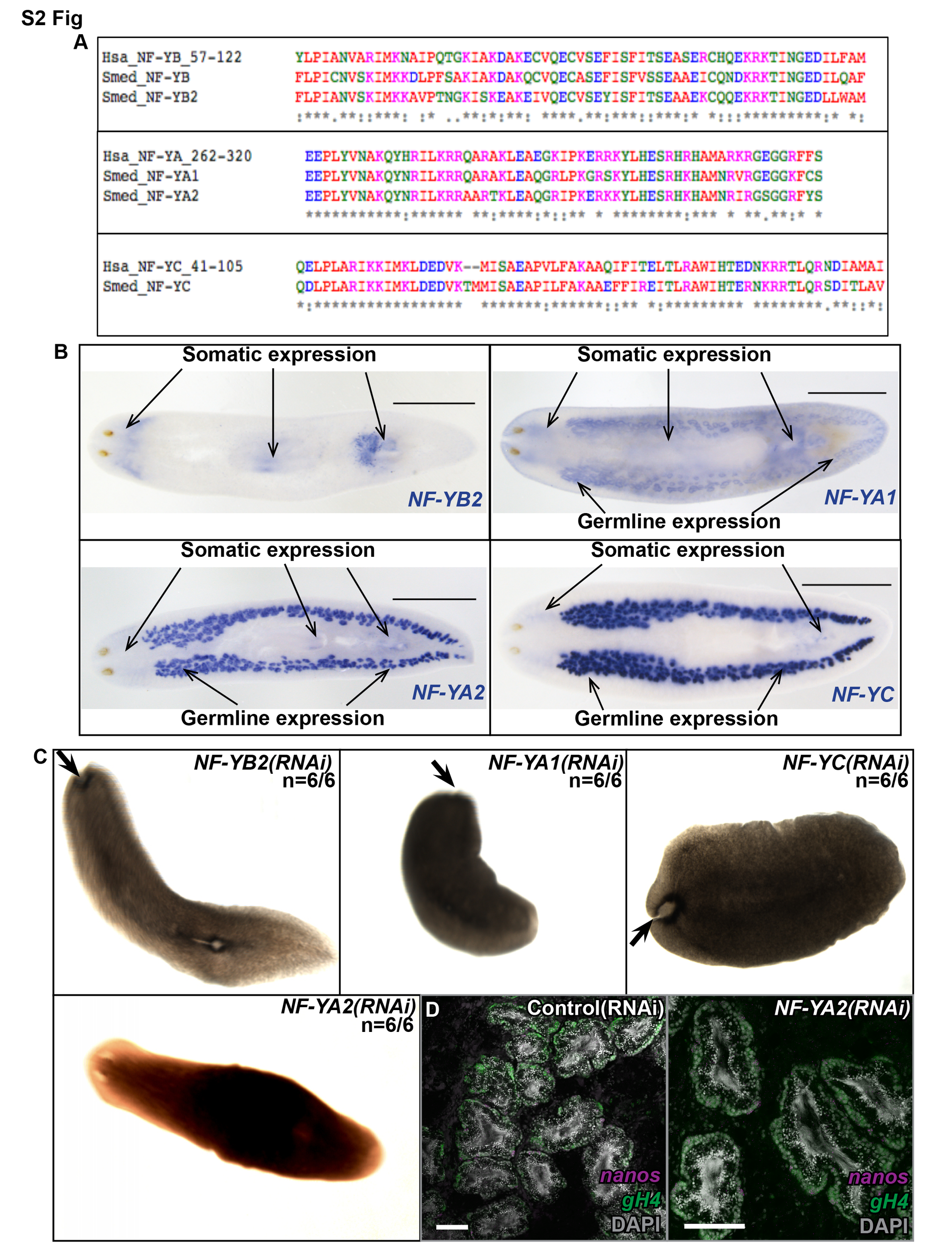

Supplement: S2 Fig — (A) ClustalW analysis of the human and planarian NF-Y complex members showing the highly conserved domains. (B) NF-YB2 transcript is expressed in somatic tissues. NF-YA1, NF-YA2, and NF-YC are expressed in both the testes and the soma. Scale bars, 1 mm. (C) RNAi of NF-YB2, NF-YA1, or NF-YC results in lesions, head regression (shown with arrows), and lethality after 5 feedings of dsRNA spaced 5 days apart. NF-YA2(RNAi) animals show no somatic phenotype. (D) NF-YA2(RNAi) animals show no loss of germ cells following 6 feedings of dsRNA. Scale bars, 50 μm. (TIF) [file pgen.1006109.s002.tif]

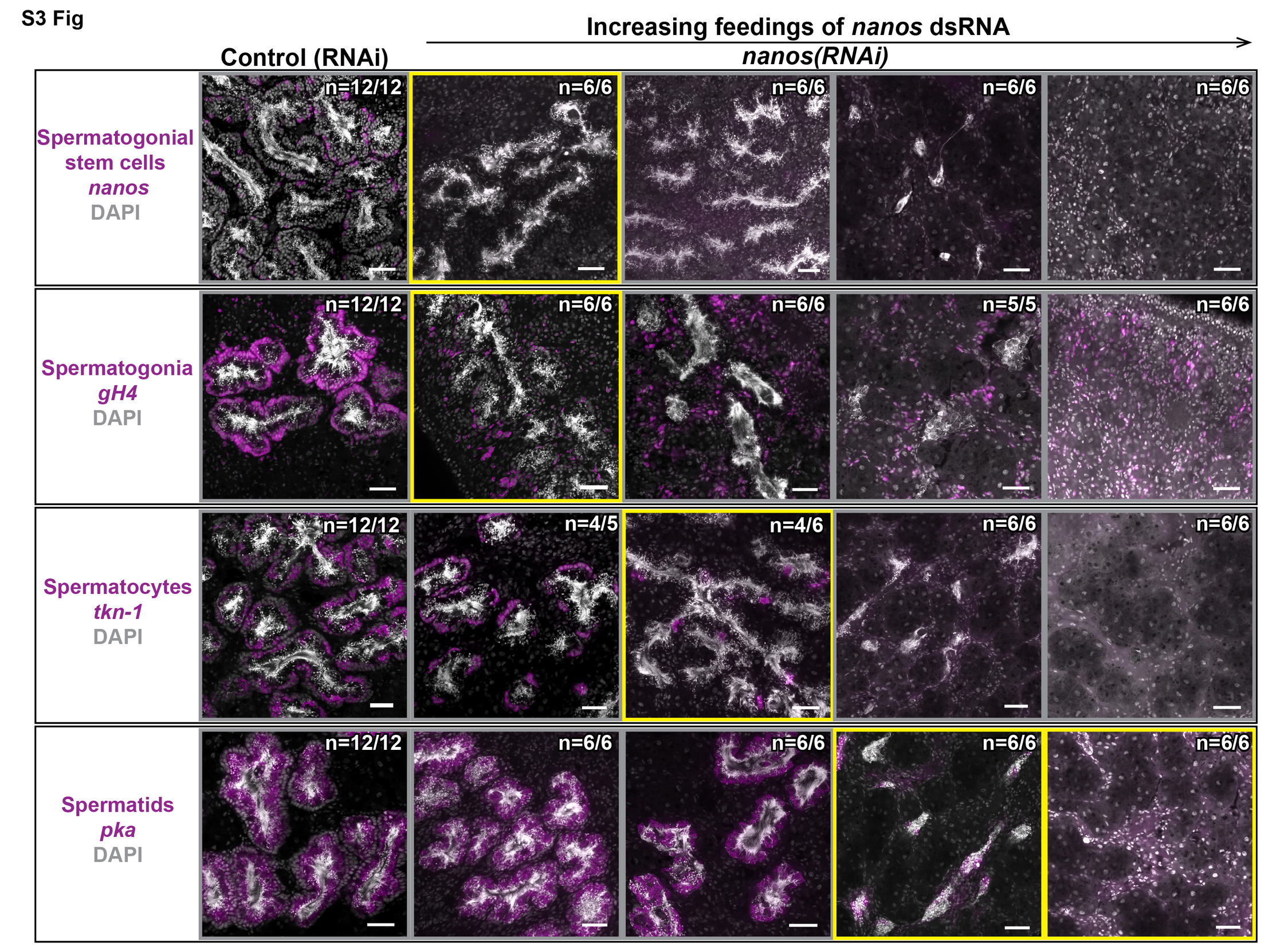

Supplement: S3 Fig — Animals show an initial loss of SSCs and spermatogonia followed by the more differentiated cells of the testes. Animals were fixed following 2, 4, 6, and 8 feedings, with 4–5 day intervals between feedings. There are subtle differences between NF-YB and nanos knockdown animals. In addition to the loss of early germ cells, NF-YB(RNAi) animals also show the loss of mature sperm to varying degrees. After 4 feedings of dsRNA, the most differentiated stage present in NF-YB(RNAi) animals is round spermatids. nanos(RNAi) animals do not show loss of spermatozoa during the initial stages of RNAi. The nanos(RNAi) phenotype also manifests faster. Scale bars, 50 μm. (TIF) [file pgen.1006109.s003.tif]

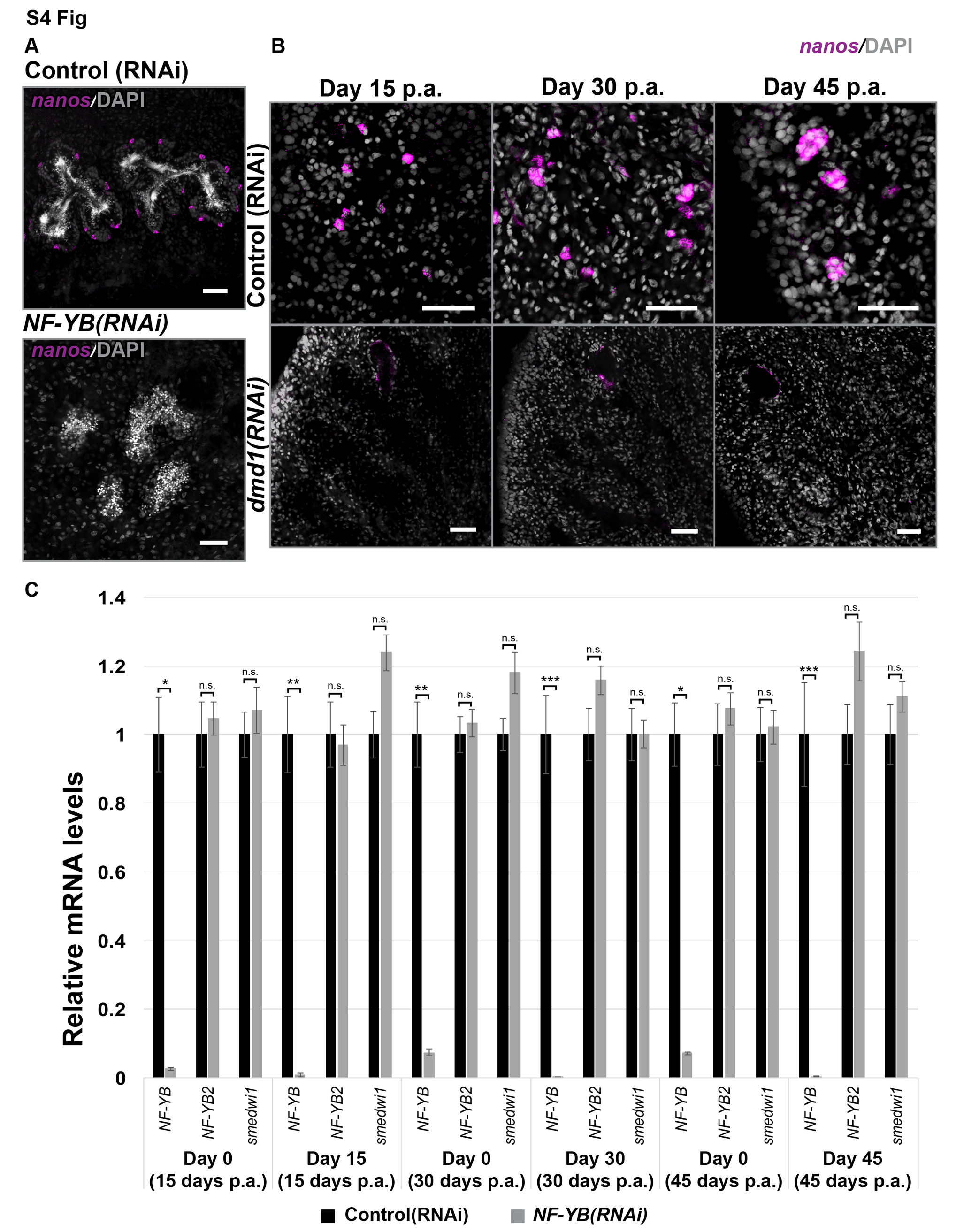

Supplement: S4 Fig — (A) Following 6 feedings of dsRNA, nanos was not detected in the testes of NF-YB(RNAi) animals. (B) dmd1(RNAi) animals do not respecify their male germ cells. Scale bars, 50 μm. (C) qRT-PCR to measure the levels of the NF-YB transcript (to determine the efficiency of knockdown), NF-YB2 transcript (to ensure specificity of NF-YB knockdown), and smedwi1 transcript (to determine if the somatic stem cells/neoblasts are perturbed following NF-YB knockdown). RNA extraction was done immediately following amputation (Day 0), and at timepoints when head regenerates were fixed for nanos in situ hybridization (Days 15, 30, or 45). Unpaired, parametric two-tailed T-test with Welch’s correction was performed on all samples. NF-YB(RNAi) animals showed significant reduction in NF-YB mRNA levels (*** = P value 0.0001–0.001; ** = P value 0.001–0.01; * = P value 0.01–0.1; n.s. = not significant). (TIF) [file pgen.1006109.s004.tif]

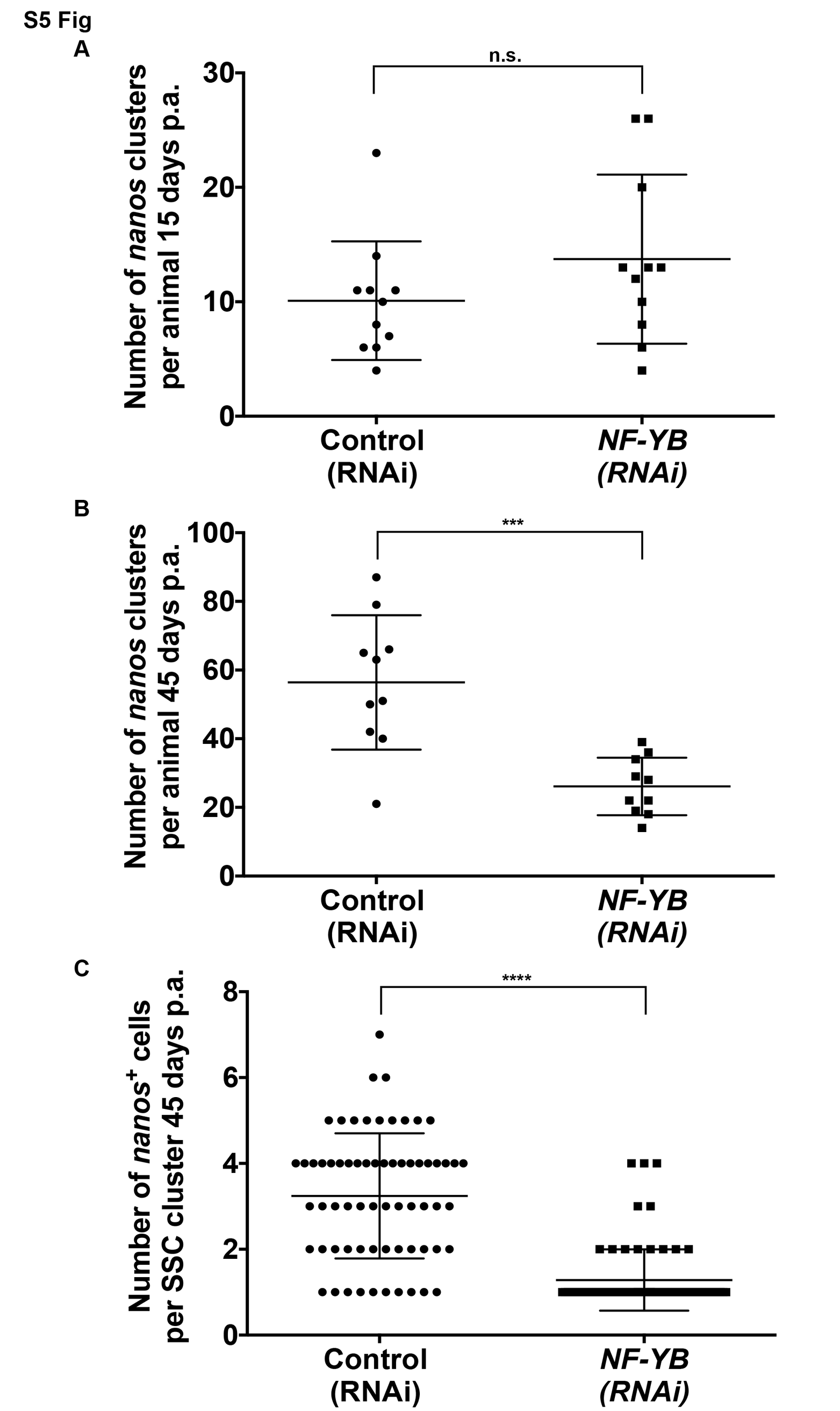

Supplement: S5 Fig — (A) 15 days post amputation (p.a.) control and NF-YB(RNAi) animals showed 10.1 ± 1.6 (n = 11/11) and 13.7 ± 2.2 (n = 11/11) SSCs respectively. The difference was not significant. (B) 45 days p.a. control animals (56.4 ± 6.2, n = 10/10) showed significantly (P<0.05) higher number of SSC clusters than NF-YB(RNAi) animals (26.1 ± 2.7, n = 10/10). (C) 45 days p.a., the number of nanos+ cells per SSC cluster was significantly (P<0.05) higher in control animals (3.2 ± 0.2, n = 66 from 10 animals) compared to NF-YB(RNAi) animals (1.3 ± 0.1, n = 74 from 10 animals). Scatter plots show mean with SD. Unpaired parametric two-tailed T-test with Welch’s correction was performed on all samples to determine significance (**** = P value <0.0001; *** = P value 0.0001–0.001; n.s. = not significant). (TIF) [file pgen.1006109.s005.tif]

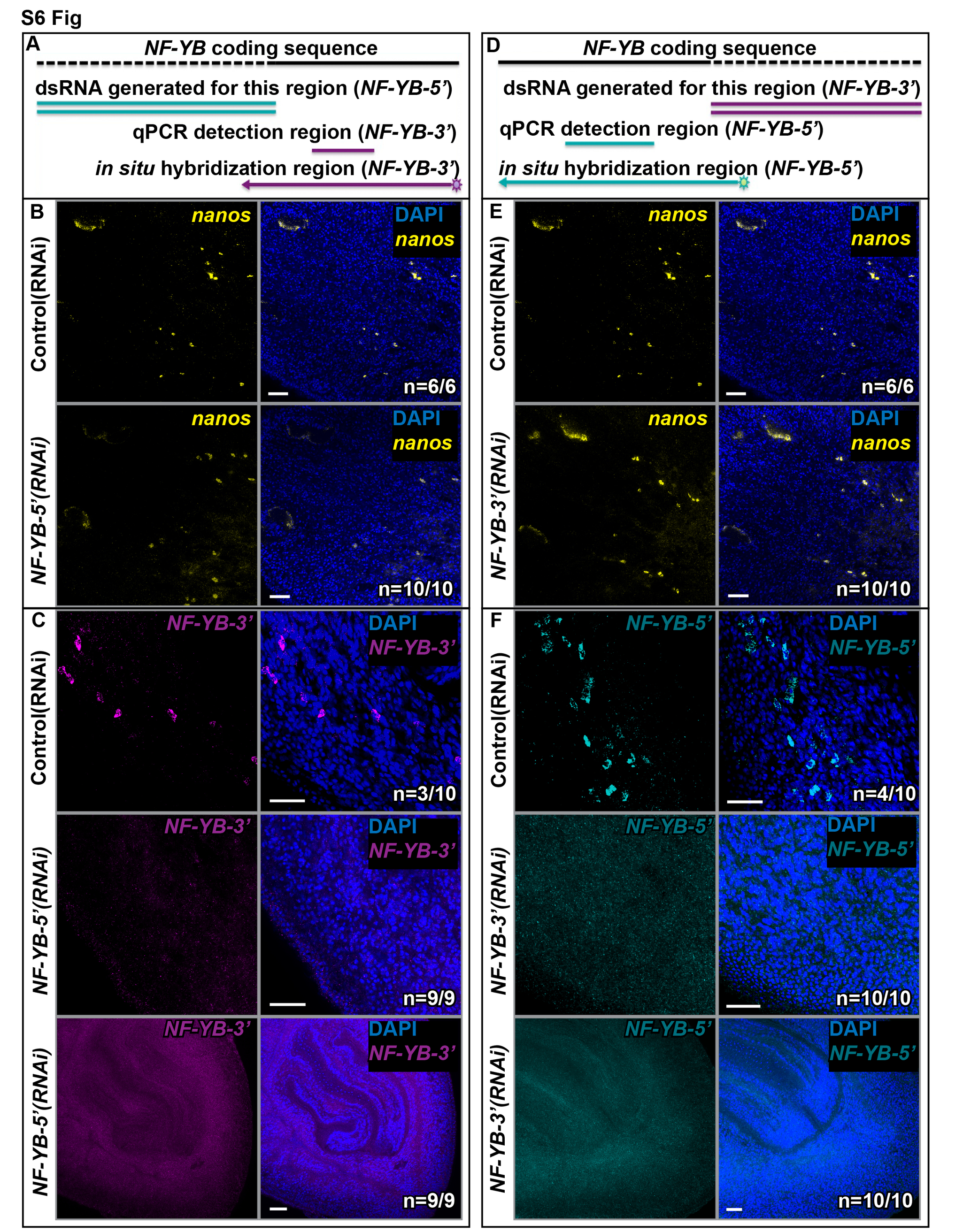

Supplement: S6 Fig — This experiment was performed to demonstrate that two halves of the NF-YB transcript can each knock down NF-YB mRNA and nanos+ SSCs are respecified in either knockdown experiment. (A) Experimental schematic. The experiment for de novo respecification of germ cells was repeated using dsRNA corresponding to the 5’ end of the NF-YB coding sequence as template. In situ hybridization was used to detect NF-YB and nanos mRNAs. A riboprobe corresponding to the 3’ end of NF-YB coding sequence was generated and used for FISH. (B) Control (RNAi) and NF-YB-5’(RNAi) animals show nanos expression following regeneration. (C) Control (RNAi) animals show expression of NF-YB, NF-YB-5’(RNAi) animals do not. Bottom panel–low magnification view of the hatchling with additional exposure showing the inability to detect NF-YB transcript throughout the animal. (D-F) The above experiment was also performed using the 3’ end of the NF-YB transcript. Scale bars, 50 μm. (TIF) [file pgen.1006109.s006.tif]

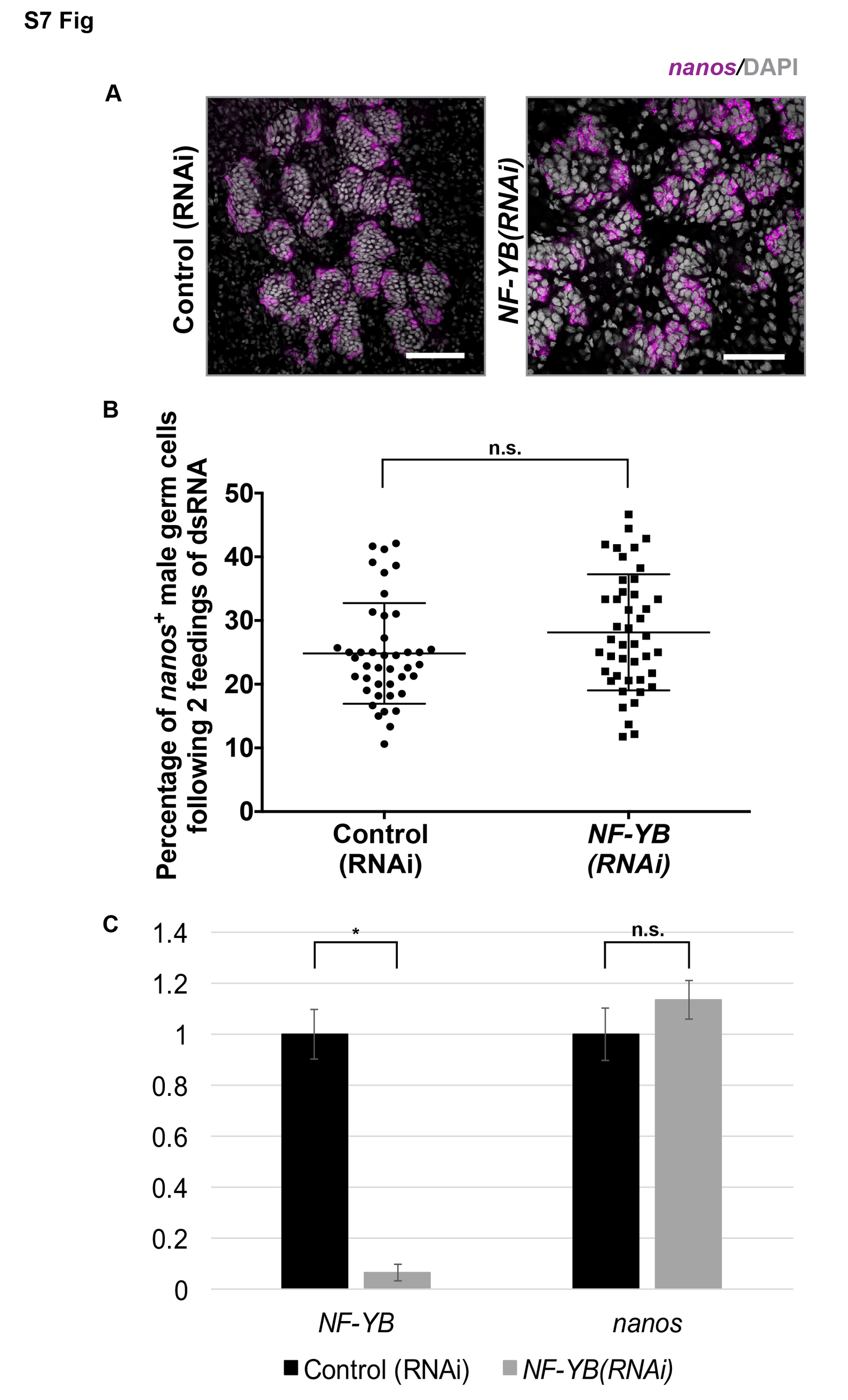

Supplement: S7 Fig — (A) Following 2 feedings of dsRNA (n = 6/6), NF-YB(RNAi) animals exhibit robust nanos labeling. From multiple prior RNAi experiments, we know that loss of nanos+ cells in NF-YB(RNAi) animals occurs only following 4–6 feedings of dsRNA. Scale bars, 50 μm. (B) We quantified SSCs in control and NF-YB(RNAi) animals to ensure that the reduced PH3S10 labeling was not due to fewer nanos+ cells in NF-YB(RNAi) animals. Following 2 feedings of dsRNA (6 animals each, 4–8 testis lobes per animal), percentage of nanos+ cells per testis lobe in NF-YB(RNAi) animals (28.2 ± 1.4, n = 44) was not significantly different (P<0.05) from control (RNAi) animals (24.8 ± 1.2, n = 42). Unpaired parametric T-test with Welch’s correction was performed. Scatter plot shows mean with SD. (C) qRT-PCR assay showing that nanos mRNA levels were unaffected following 2 feedings of NF-YB dsRNA. Unpaired parametric two-tailed T-test with Welch’s correction was performed to determine significance (* = P value 0.01–0.1; n.s. = not significant). (TIF) [file pgen.1006109.s007.tif]

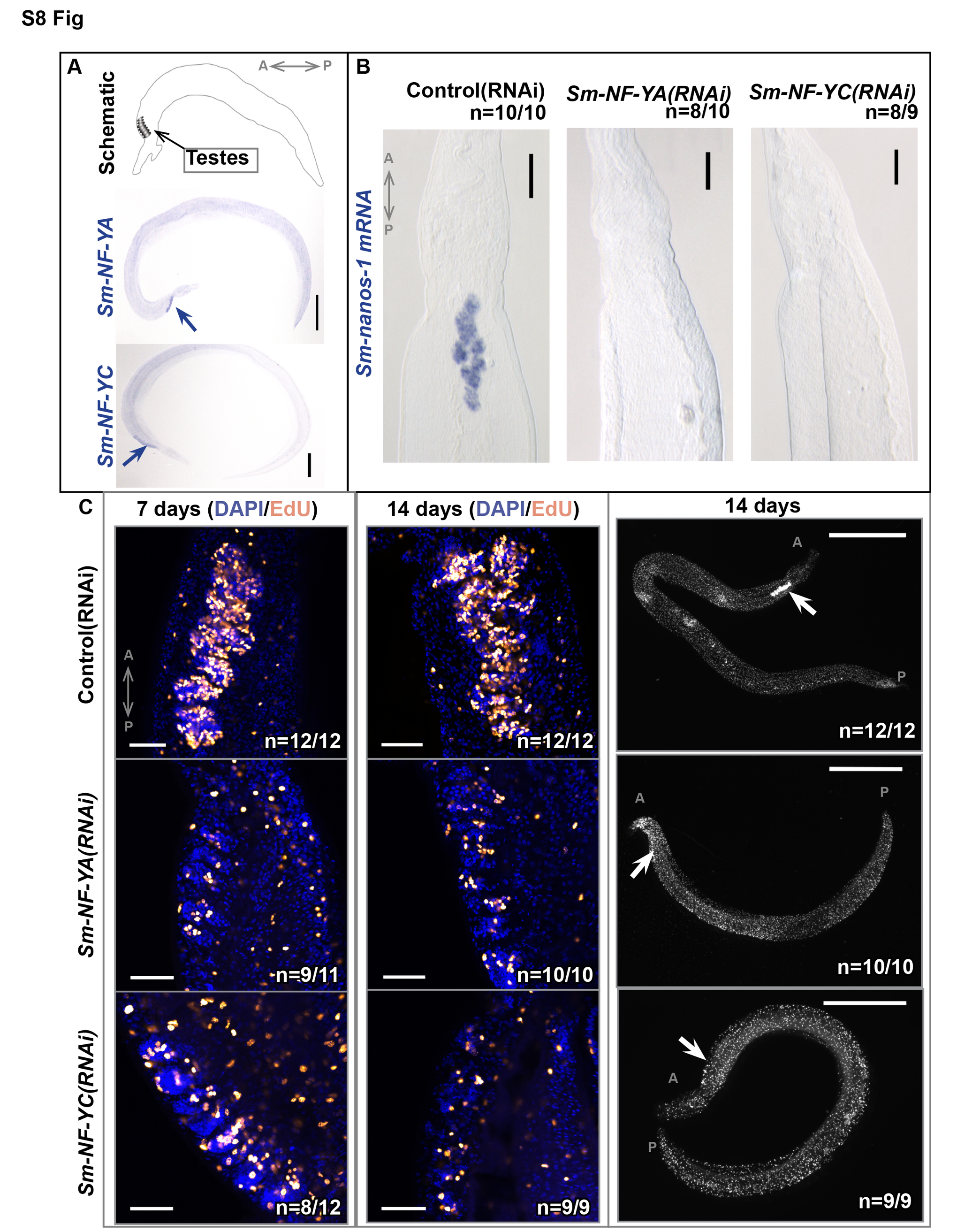

Supplement: S8 Fig — (A) Illustration of male S. mansoni depicting the location of the testes and whole-mount in situ hybridization (WISH) in male schistosomes showing Sm-NF-YA and Sm-NF-YC expression in testes. Scale bars, 1 mm. (B) Magnified view of Sm-nanos-1 expression in control (RNAi), Sm-NF-YA(RNAi), and Sm-NF-YC(RNAi) animals. Sm-nanos-1 expression is not detected in Sm-NF-YA(RNAi), and Sm-NF-YC(RNAi) animals. Scale bars, 1 mm. (C) Left and middle panels show high magnification view of the testes in control (RNAi), Sm-NF-YA(RNAi), and Sm-NF-YC(RNAi) animal at early and late KD time points. Scale bars, 50 μm. Right panel shows whole-mount images showing reduction or loss of EdU labeling in the testes in Sm-NF-YA(RNAi) and Sm-NF-YC(RNAi) animals. Scale bars, 1 mm. (TIF) [file pgen.1006109.s008.tif]
