# Supplementary material for: NF-YB Regulates Spermatogonial Stem Cell Self-Renewal and Proliferation in the Planarian Schmidtea mediterranea
Source: PLoS Genet. 2016 Jun 15;12(6):e1006109. doi: 10.1371/journal.pgen.1006109 (PMC4909293; doi:10.1371/journal.pgen.1006109)
Supplement: S1 Table — (DOCX) [file pgen.1006109.s009.docx]

**S1 Table. Raw data for quantification performed in the manuscript**

**Quantification of *NF-YB(RNAi)* phenotype in homeostasis**

| **Germ cell marker/type** | **Days of RNAi (number of feedings)** | **Number of lobes showing normal expression of germ cell marker** | **Total number of testis lobes counted** | **Percentage** |
| --- | --- | --- | --- | --- |
| ***nanos*/SSCs** | 14 days (4 feedings) | 28 | 60 | 46.7 |
|  | 23 days (6 feedings) | 19 | 60 | 31.7 |
|  | 32 days (8 feedings) | 0 | 60 | 0 |
|  | 42 days (10 feedings) | 0 | 40 | 0 |
| ***gH4*/SSCs, spermatogonia** | 14 days (4 feedings) | 32 | 60 | 53.3 |
|  | 23 days (6 feedings) | 16 | 60 | 26.7 |
|  | 32 days (8 feedings) | 0 | 60 | 0 |
|  | 42 days (10 feedings) | 0 | 40 | 0 |
| ***tkn-1*/ spermatocytes** | 14 days (4 feedings) | 50 | 60 | 83.3 |
|  | 23 days (6 feedings) | 38 | 60 | 63.3 |
|  | 32 days (8 feedings) | 19 | 60 | 31.7 |
|  | 42 days (10 feedings) | 0 | 40 | 0 |
| ***pka*/spermatids** | 14 days (4 feedings) | 60 | 60 | 100 |
|  | 23 days (6 feedings) | 41 | 50 | 82 |
|  | 32 days (8 feedings) | 28 | 60 | 46.7 |
|  | 42 days (10 feedings) | 5 | 40 | 12.5 |
| **sperm** | 14 days (4 feedings) | 10 | 60 | 16.7 |
|  | 23 days (6 feedings) | 0 | 60 | 0 |
|  | 32 days (8 feedings) | 0 | 60 | 0 |
|  | 42 days (10 feedings) | 0 | 40 | 0 |

**Quantification of PH3S10^+^ cells in *NF-YB(RNAi)* animals**

| **Number of PH3S10 postive cells per testis lobule** | **CTRL(RNAi) - 2 feedings** | ***NF-YB(RNAi) -* 2 feedings** | **CTRL(RNAi) - 4 feedings** | ***NF-YB(RNAi) -* 4 feedings** |
| --- | --- | --- | --- | --- |
| 0 | 5 | 32 | 1 | 44 |
| 1 | 6 | 5 | 1 | 3 |
| 2 | 11 | 1 | 4 | 1 |
| >2 | 20 | 1 | 11 | 1 |
| **Number of planarians** | **n=8** | **n=8** | **n=7** | **n=8** |

**Quantification of TUNEL^+^ cells in *NF-YB(RNAi)* animals**

| **CTRL(RNAi) - 2 feedings** | | | ***NF-YB(RNAi)* - 2 feedings** | | |
| --- | --- | --- | --- | --- | --- |
| **Total number of cells** | **Number of apoptotic cells** | **Apoptotic index** | **Total number of cells** | **Number of apoptotic cells** | **Apoptotic index** |
| 34 | 0 | 0 | 24 | 1 | 0.04 |
| 36 | 0 | 0 | 36 | 0 | 0 |
| 45 | 0 | 0 | 22 | 0 | 0 |
|  |  |  |  |  |  |
|  |  |  |  |  |  |
| **CTRL(RNAi) - 4 feedings** | | | ***NF-YB(RNAi)* - 4 feedings** | | |
| **Total number of cells** | **Number of apoptotic cells** | **Apoptotic index** | **Total number of cells** | **Number of apoptotic cells** | **Apoptotic index** |
| 140 | 0 | 0 | 75 | 14 | 0.19 |
| 128 | 0 | 0 | 90 | 8 | 0.09 |
| 121 | 0 | 0 | 110 | 14 | 0.13 |
