# Supplementary material for: NF-YB Regulates Spermatogonial Stem Cell Self-Renewal and Proliferation in the Planarian Schmidtea mediterranea
Source: PLoS Genet. 2016 Jun 15;12(6):e1006109. doi: 10.1371/journal.pgen.1006109 (PMC4909293; doi:10.1371/journal.pgen.1006109)
Supplement: S2 Table — (DOCX) [file pgen.1006109.s010.docx]

**S2 Table. Primer sequences**

| Smed-NF-YB-forward cloning primer | TTGCAACGTTTCGAAAATAATG |
| --- | --- |
| Smed-NF-YB-reverse cloning primer | AAAGGAGTCTTCATCGGTTGAT |
| Smed-NF-YB5'-forward cloning primer | ATGGACGATAACATGAATAC |
| Smed-NF-YB5'-reverse cloning primer | TGCCTCTCTATAAGTTTGCA |
| Smed-NF-YB5'-forward qRT-PCR primer | CCTGCCGATTTGCAACGTTTCGA |
| Smed-NF-YB5'-reverse qRT-PCR primer | TCTGCTGCTTCGCTGCTCACA |
| Smed-NF-YB3'-forward cloning primer | GGAGACCTTGCAGAACTTTTTGC |
| Smed-NF-YB3'-reverse cloning primer | TTATAATGCTTCATCCATA |
| Smed-NF-YB3'-forward qRT-PCR primer | GCCTTGGTCAATGGCGCCAGTA |
| Smed-NF-YB3'-reverse qRT-PCR primer | TCGGTCAACGGGAGCATGGAA |
| Smed-NF-YB2-forward cloning primer | ACCAAGTCCAGCAAGGTGAT |
| Smed-NF-YB2-reverse cloning primer | ACCGGCTTCACTCTGAACAT |
| Smed-NF-YA1-forward cloning primer | GGAATCCAAACTAGTGATGGAAATA |
| Smed-NF-YA1-reverse cloning primer | TCTGAGTTGTGCTTTGAGATGA |
| Smed-NF-YA2-forward cloning primer | TTTAACAACCAGTTCCGATGG |
| Smed-NF-YA2-reverse cloning primer | CGCTAGAGACACCAATTCTCG |
| Smed-NF-YC-forward cloning primer | CGTGAAATCACATTGAGAGCA |
| Smed-NF-YC-reverse cloning primer | TTGGTCTGGTTTCCACCAAT |
| Smed_dmd-1-forward qRT-PCR primer | GACGTCAAACCGAATTTACTGA |
| Smed_dmd-1-reverse qRT-PCR primer | TTTCACCGGCAACAACTG |
| Smed_nanos-forward qRT-PCR primer | CAAGGACAAATGTTGCCTGTA |
| Smed_nanos-reverse qRT-PCR primer | CAACCCATCGATCCAACTCT |
| Smed_NF-YB2-forward qRT-PCR primer | AATATGGACCACCAAGTCCAG |
| Smed_NF-YB2-reverse qRT-PCR primer | GTTCACGAAGTGGCGAAAGT |
| Smed_smedwi-forward qRT-PCR primer | GAGCTGGGGGATGTGTATTG |
| Smed_smedwi-reverse qRT-PCR primer | CACCAAAACGATGAGCTGTG |
| Sm-nanos-1-forward cloning primer | TGATATCGAAATCGTCCAGACA |
| Sm-nanos-1-reverse cloning primer | TTGTATCGGTTCCAACAAACC |
| Sm-NF-YB-forward cloning primer | TGTGGACTATCAAGCAGGTGA |
| Sm-NF-YB-reverse cloning primer | TGGAGTCGATACGGAATGAA |
| Sm-NF-YA-forward cloning primer | CCGGCCCAAACTATTCTACA |
| Sm-NF-YA-reverse cloning primer | AGCATGCTTATGCCTGGACT |
| Sm-NF-YC-forward cloning primer | GTTGGCCGACGATGTCCT |
| Sm-NF-YC-reverse cloning primer | TTTCTCATAAATGGATTACTCAAGTG |
